# The Potential Role of Combination Pharmacotherapy to Improve Outcomes of Pediatric Obesity: A Case Report and Discussion

**DOI:** 10.3389/fped.2018.00361

**Published:** 2018-11-27

**Authors:** Claudia K. Fox, Aaron S. Kelly

**Affiliations:** Department of Pediatrics, Center for Pediatric Obesity Medicine, University of Minnesota, Minneapolis, MN, United States

**Keywords:** pharmacotherapy, obesity, pediatric, medication, topiramate, phentermine

## Abstract

There is a gap in treatment modalities for pediatric patients with obesity for whom lifestyle modification therapy, on the one hand, may be insufficient to meaningfully reduce BMI, and bariatric surgery, which on the other hand, may not be indicated, available or desired. Although pharmacotherapy may help fill this treatment void, there is a paucity of FDA-approved medications indicated for pediatric obesity and further, most are single agents with only modest mean treatment effects. In contrast, combination pharmacotherapy, such as phentermine/topiramate, appears to offer greater weight loss efficacy in adults and may prove to be superior to monotherapy for pediatric patients as well. This case report describes the clinical management of severe obesity in a 10 year old girl with lifestyle modification therapy and subsequent addition of first topiramate and later phentermine. Using the case as a platform, the current state of pharmacotherapy for pediatric obesity will be described thereby highlighting the limited efficacy of single agents. Additionally, the biological rationale for combination pharmacotherapy, including potential mechanisms which may account for the poor response to single agents, will be discussed.

## Background

Lifestyle modification therapy (LSMT), which consists of dietary and physical activity counseling supported by behavioral strategies, is the cornerstone of obesity treatment. However, multiple studies indicate that for youth, particularly adolescents, with severe obesity, LSMT is often insufficient for achieving clinically significant and durable BMI reduction (1–4). Some data suggest that fewer than 2% of youth with severe obesity are able to achieve and *maintain* clinically-meaningful reduction in BMI.^1^

The challenge of weight loss maintenance stems from both behavioral and physiological responses to the reduced energy stores and negative energy balance. While long-term adherence to behavior changes tends to wane with time, the drive behind the waning effect is primarily physiological in nature. As fat stores decrease, fasting leptin and insulin decrease, thereby conveying a message of energy depletion to the hypothalamus resulting in increased hunger sensation. Additionally, cross talk between the hypothalamus and “non-homeostatic” hedonic and cognitive brain regions leads to a heightened sense of food palatability. Concurrently, total energy expenditure decreases due to decreased body mass and enhanced metabolic efficiency (5, 6) This process is termed “metabolic adaptation” and the net result is weight regain.

Obesity pharmacotherapy may address some of the physiological adaptations and counter-regulatory mechanisms that contribute to weight regain primarily via their effect on appetite and hunger. In this case report, we describe the use of medications, specifically topiramate and phentermine, for weight regain after success with LSMT in a 10 year old girl with severe obesity.

## Case presentation

A 10 year old white girl with severe (class 3: BMI ≥140% of the 95th percentile for age and sex) obesity and otherwise normal development presented to the Pediatric Weight Management Clinic with her mother. The mother reported that the patient had been at the 75th percentile for height and weight for most of the patient's life but she experienced a “20 to 30 pound” weight gain over the past year. The mother further explained that this recent weight gain coincided with treatment of seasonal allergies with montelukast and she wondered if this may have been the cause of the weight increase. The patient had no prior weight loss attempts.

The patient was born full term, weighing 3.18 kg. The mother's pregnancy was uncomplicated, as was the patient's newborn course. Aside from seasonal allergies, the patient was healthy. She had no history of hospitalizations, surgeries, or mental health concerns. She was not taking any medications.

The patient was eating regularly-spaced meals consisting primarily of highly processed foods and simple carbohydrates (e.g., pastries for breakfast, potatoes with cheese for dinner). The family was eating fast food three times per week on average. The patient endorsed having a big appetite and feeling hungry all the time. She was eating while watching TV and when bored. She denied binge eating, loss of control eating, emotional eating, sneaking/hiding food, or eating during the night. Her physical activity was limited to gym class at school three times per week.

The patient was living with her mother and her mother's partner. The patient's parents divorced when she was very young and the mother's partner had been living with them since the patient was a toddler. The patient saw her biological father rarely. She had no siblings. She was attending fourth grade and enjoyed reading and writing. The mother and her partner worked full-time and the patient was cared for by a baby sitter after school a few times per week. They had no food insecurity. The family history was notable for obesity in both biological parents and type 2 diabetes in the maternal grandmother.

The patient's review of systems was negative. She reached menarche several months prior to presentation. On physical examination, her weight was 70.31 kg (155 lbs.), height was 142 cm (4'8”), and BMI was 34 kg/m^2^ (145% of the 95th percentile). Her blood pressure was 105/65 mmHg and pulse was 74 beats per minute. Her physical examination was normal. The results of her fasting labs were: total cholesterol 176 mg/dL (normal: < 170 mg/dL), HDL-c 49 mg/dL (>45 mg/dL), LDL-c 96 mg/dL (< 110 mg/dL), triglycerides 157 mg/dL (< 90 mg/dL), ALT 27 (< 50 U/L), AST 29 (< 50 U/L), glucose 98 mg/dL (70-99 mg/dL), and HbA1c 5.5% (0-5.6%). Her Pediatric Symptom Checklist (routinely obtained in the Pediatric Weight Management Clinic) score was 8 (> 28 is considered abnormal).

The patient and family were started on a program of lifestyle modification therapy and responded particularly well with decreasing fast food consumption and liquid calories. Further, the patient started bringing her lunch to school instead of eating the school fare and was able to keep a food log almost daily. The patient's physical activity, however, continued to be limited. Over the course of 5 months, the patient's BMI decreased 5 units (15%), from 34 kg/m^2^ to 29 kg/m^2^ (145% of the 95th percentile to 125% of the 95th percentile).

**Figure 1:**
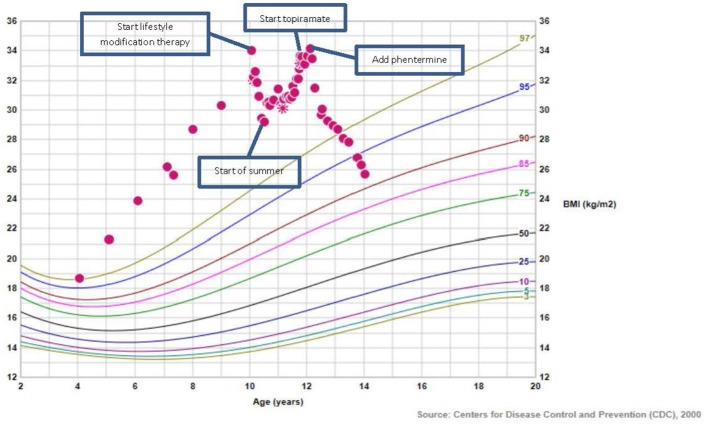
BMI growth chart of patient.

At the end of the 5 month period, coinciding with the end of the school year and beginning of summer vacation, the patient's sleep/wake cycle became irregular. Because she did not like the hot weather, she chose to stay inside all day. Her mother left prepared meals for the patient to encourage healthy eating while mom was at work. Despite this, the patient's BMI began to trend upward from 29 kg/m^2^ to 31 kg/m^2^ over the summer months. Upon school resuming in the fall, the patient's sleep/wake cycle normalized and eating behaviors and patterns improved, returning to those of the previous school year. The patient's BMI stabilized for a few months but then increased further. The patient expressed frustration because she believed that she was eating well, which was indeed reflected in her daily food logs. She continued to attend monthly visits with the Pediatric Weight Management Clinic dietician, psychologist, and pediatrician with specialized training in obesity medicine. Yet, the patient's BMI continued to increase such that by 2 years after her initial appointment, the patient's BMI returned to baseline (135% of the 95th percentile) (see Figure 1).

Suspecting that metabolic adaptation was causing the patient's weight rebound, adjunct pharmacotherapy was recommended. Orlistat was considered but not started because of concern about gastrointestinal side effects and lack of insurance coverage. Metformin may have been another reasonable option but the patient's fasting glucose and HbA1c were in the normal range and she did not have acanthosis nigricans on physical examination which would have suggested insulin resistance. She was ultimately started on topiramate 75 mg daily in addition to ongoing LSMT. She and her mother were cautioned that although topiramate is not FDA-approved for the indication of obesity (in children or adults), multiple studies have demonstrated clinically-meaningful weight loss efficacy in adults. Additionally, it was explained that the side effect profile in children is well established stemming from its use for epilepsy treatment.

After 4 months of treatment with topiramate, the patient's BMI trajectory plateaued yet was not decreasing as was desired. Recognizing that the combination of topiramate and phentermine is the most effective weight loss medication currently available for adult obesity, phentermine 15 mg daily was added to the topiramate 75 mg daily. The patient and mother were informed that phentermine is FDA-approved only for individuals older than 16 years and for “short-term use.” With combination treatment for ~22 months, the patient experienced good BMI reduction, from 34.1 to 25.7 kg/m^2^. Her blood pressure and heart rate were monitored regularly and though her blood pressure did not increase, her heart rate increased slightly from 60 to 70 s, in line with the mechanisms of action of phentermine (stimulant-like effects). Later, the patient reported that she was experiencing some “memory” issues but noted no change in her academic performance. Although it seemed unusual for this type of symptom to emerge 10 months after starting topiramate, the topiramate dose was decreased from 75 to 50 mg daily and the memory issues resolved. Written informed consent was obtained from the parent of the patient for the publication of this case report.

## Discussion

Although the patient in the case presentation was able to impressively reduce her BMI via LSMT by nearly 15% over the course of 5 months, she was unable to maintain her BMI reduction despite her efforts in sustaining the dietary and physical activity behaviors that allowed her to reduce her BMI initially. This is not unexpected and highlights the effect of metabolic adaptation and the need for obesity pharmacotherapy to help sustain weight loss in many patients.

In the pediatric population there are only two FDA-approved medications indicated for obesity: orlistat and phentermine. Orlistat, a lipase inhibitor (which does not necessarily address physiological adaptations to weight loss), is FDA-approved for patients ≥12 years of age. In the largest study examining the effect of orlistat on BMI change in youth, 539 adolescents with obesity were randomized to either LSMT plus orlistat (120 mg three times daily) or placebo for 1 year. At the end of the study, BMI decreased 0.55 kg/m^2^ in the orlistat group and increased 0.31 kg/m^2^ in the placebo group. This translates into a treatment effect of ~3% decrease in BMI from baseline. Further, there were no clinically significant changes in blood pressure, fasting lipids, insulin, or glucose tolerance. Mild to moderate gastrointestinal adverse events (most commonly fatty/oily stool) occurred in 9%-50% of the orlistat group compared to 1–13% of the placebo group (7).

Phentermine, a sympathomimetic (norepinephrine reuptake inhibitor), was approved by the FDA in 1959 for individuals >16 years of age for “short term use” and is currently the most widely prescribed medication for the treatment of obesity in adults (8). Although there are no randomized, controlled studies of this medication in adolescents that are more than 1-month in duration, a retrospective chart review of adolescents with severe obesity treated in a pediatric weight management clinic reported the six-month outcomes of phentermine plus LSMT on BMI. In this study, 25 adolescents with severe obesity treated with LSMT plus phentermine (most commonly 15 mg daily) were compared to 274 age- and BMI-matched adolescents treated with LSMT alone. At six months, the LSMT plus phentermine group decreased their BMI by 1.6 kg/m^2^ [95% CI (−2.8 kg/m^2^, −0.4 kg/m^2^); *p* = 0.011] more than the LSMT alone group, translating into a treatment effect of 4% [(−7.1%, −1.0%); *p* = 0.009] decrease in BMI. There were no significant differences in blood pressure between groups, though heart rate was higher (but not statistically significant) in the phentermine group (9). These findings of no increase in blood pressure with phentermine are consistent with what has been found in adults. In adults, phentermine is not associated with increase in heart rate either (10). Further, phentermine abuse, psychological dependence, and withdrawal do not occur in adult patients treated with phentermine for obesity, even after years of treatment (11).

Among the other medications that have been studied for the indication of obesity in the pediatric population, metformin, exenatide, and topiramate have the most data. Metformin is FDA approved for type 2 diabetes in children ≥ 10 years of age and has modestly favorable weight loss properties. A meta-analysis of 14 randomized clinical trials examining metformin for pediatric obesity demonstrated an overall effect size of−1.16 kg/m^2^ (12). Total cholesterol, but not other lipid outcomes or blood pressure, decreased slightly more with metformin compared with the control groups. Gastrointestinal adverse events were reported in 26% of the metformin group and 13% of the control group.

Exenatide, a glucagon-like peptide-1 receptor agonist, belongs to the class of incretins that also includes liraglutide which is FDA approved for adult obesity. In adolescents, two pilot placebo-controlled studies examined the effect of exenatide on BMI. In the first, 12 adolescents were randomized to exenatide 5 mcg twice daily or placebo for 3 months. The exenatide group decreased their BMI by 0.9 kg/m^2^ and the control group increased their BMI by 0.84 kg/m^2^. The treatment effect was −4.92% [(−8.61, −1.23); *p* = 0.009] BMI reduction (13). In the second, 26 adolescents were randomized and exenatide elicited a 2.7% [(−5.02,−0.37); *p* = 0.03] BMI reduction at 3 months. There were no significant changes in blood pressure, heart rate, lipids, or glucose. Nausea was the most common adverse event reported in 62% of the exenatide group and 31% of the placebo group (14).

Finally, topiramate, an antiepileptic agent that is FDA approved for seizures in children ≥ 2 years of age, has been extensively studied for the treatment of adult obesity (15). For youth, a retrospective chart review of 28 adolescents treated with LSMT plus topiramate (most commonly 75 mg daily) in a pediatric weight management clinic demonstrated a 4.9% [(−7.1, −2.8), *p* < 0.001] BMI reduction at 6 months (16). In a double blind pilot study, 30 adolescents with severe obesity completed four weeks of meal replacement therapy followed by randomization to 24 weeks of either topiramate 75 mg daily or placebo. There was a modest reduction in BMI (~2%) that was not statistically significant between the topiramate group and the placebo group. The most common side effect was paresthesia and there were no adverse changes in cognitive function measured by highly sensitive instruments (17).

The outcomes of studies examining single medications, in contrast to combination medications, for the treatment of obesity in both adults and children demonstrate only modest effects of 3–5% weight or BMI reduction. This is true of the patient in the case. In fact, the patient in the case only experienced a suspension in her BMI increase, and not a BMI decrease, with topiramate monotherapy. It was not until phentermine was added to the topiramate that her BMI decreased significantly.

Combination pharmacotherapy may have advantages over monotherapy for several reasons. One is that there are redundant processes involved in the control of body weight. By employing medications with different mechanisms of action, the ability to effect more than one of these processes is enhanced thereby increasing the opportunity for a greater impact on adiposity. Second, employing more than one medication at a time allows for the possibility of using lower therapeutic doses of each of the medications which may mitigate side effects. This is particularly relevant to the pediatric population given the long duration of treatment. For example, the weight loss effects of topiramate is dose dependent but neurocognitive side effects, such as cognitive dulling and fatigue, tend to emerge above doses of 100 mg daily (15). This may limit its utility as a monotherapy. By combining a moderate dose of topiramate (i.e., <100 mg) with another weight loss medication that has a different side effect profile, such as phentermine, maximum weight loss effect may be achieved with limited adverse effects. Indeed, the combination of phentermine plus topiramate is the most effective weight loss medication currently FDA-approved for adult obesity.

## Conclusion

Severe pediatric obesity is a serious and chronic disease. For many patients, LSMT is insufficient for achieving clinically significant and durable BMI reduction because LSMT does not address the biological underpinnings of this disease. Pharmacotherapy may hold promise as a safe and effective adjunct tool for the management of severe obesity in the pediatric population, yet more clinical trials are needed. Further, as in adults, combination medications, in contrast to monotherapy, may prove to be more advantageous.

## Ethics statement

Patient's guardian gave written consent for case report to be published.

## Author contributions

CF and AK contributed to the conception of this case study. CF wrote the initial draft. Both contributed to manuscript revision, read, and approved the submitted version.

### Conflict of interest statement

CF receives research support from Novo Nordisk. AK serves as a consultant for Novo Nordisk, Orexigen, and Vivus.
